# Factors associated with study attrition in a pilot randomised controlled trial to explore the role of exercise-assisted reduction to stop (EARS) smoking in disadvantaged groups

**DOI:** 10.1186/s13063-016-1641-5

**Published:** 2016-10-27

**Authors:** T. P. Thompson, C. J. Greaves, R. Ayres, P. Aveyard, F. C. Warren, R. Byng, R. S. Taylor, J. L. Campbell, M. Ussher, S. Michie, R. West, A. H. Taylor

**Affiliations:** 1 Plymouth University Peninsula School of Medicine and Dentistry, Plymouth, UK; 2 University of Exeter Medical School, Exeter, UK; 3 Nuffield Department of Primary Care Health Sciences, University of Oxford, Oxford, UK; 4 Institute of Population Health Research, St George’s University of London, Cranmer Terrace, London, UK; 5 Research Department of Clinical, Educational and Health Psychology, University College London, 1-19 Torrington Place, London, UK; 6 Health Behaviour Research Centre, Department of Epidemiology and Public Health, University College London, Gower Street, London, UK

**Keywords:** Retention, Drop out, Physical activity, Smoking reduction, Harm reduction

## Abstract

**Background:**

Study attrition has the potential to compromise a trial’s internal and external validity. The aim of the present study was to identify factors associated with participant attrition in a pilot trial of the effectiveness of a novel behavioural support intervention focused on increasing physical activity to reduce smoking, to inform the methods to reduce attrition in a definitive trial.

**Methods:**

Disadvantaged smokers who wanted to reduce but not quit were randomised (*N* = 99), of whom 61 (62 %) completed follow-up assessments at 16 weeks. Univariable logistic regression was conducted to determine the effects of intervention arm, method of recruitment, and participant characteristics (sociodemographic factors, and lifestyle, behavioural and attitudinal characteristics) on attrition, followed by multivariable logistic regression on those factors found to be related to attrition.

**Results:**

Participants with low confidence to quit, and who were undertaking less than 150 mins of moderate and vigorous physical activity per week at baseline were less likely to complete the 16-week follow-up assessment. Exploratory analysis revealed that those who were lost to follow-up early in the trial (i.e., by 4 weeks), compared with those completing the study, were younger, had smoked for fewer years and had lower confidence to quit in the next 6 months. Participants who recorded a higher expired air carbon monoxide reading at baseline were more likely to drop out late in the study, as were those recruited via follow-up telephone calls. Multivariable analyses showed that only completing less than 150 mins of physical activity retained any confidence in predicting attrition in the presence of other variables.

**Conclusions:**

The findings indicate that those who take more effort to be recruited, are younger, are heavier smokers, have less confidence to quit, and are less physically active are more likely to withdraw or be lost to follow-up.

## Background

Participant attrition within research trials poses a threat to internal validity (attrition bias) [1], external validity (retained participants may not reflect practice) and loss of statistical power (reduced number of participants). Strategies to minimise attrition, such as knowing when and where to direct resources, may also have implications for the cost of conducting trials due to additional researcher time necessary to capture follow-up data [2]. Pilot trials can help to identify factors associated with study attrition and provide valuable information for the planning of a definitive trial, such as providing options for mode of participation (e.g. face-to-face assessments, telephone interviews, or postal questionnaires) [3] and identifying which participants are more likely to drop out and when to allow effective planning to maximise retention of participants..

It is usual for smoking cessation intervention trials to utilise intention-to-treat (ITT) analyses with an assumption that a participant lost to follow-up is still smoking (baseline observation carried forward) [4]. This assumption is problematic, as it could bias results and statistical tests in favour of an effective treatment if attrition rates are higher in the control group, as there is some evidence to suggest that those lost to follow-up in such trials may not necessarily be smoking [5–9]. Different approaches to handling missing data on smoking status at follow-up have been suggested, which may provide more reliable estimates of treatment effects [10–12]. However, all approaches rely on making assumptions about the missing data. It is therefore important to understand the factors influencing attrition to allow for more informed approaches to handling missing data, and to identify ways to minimise attrition in future smoking studies. This could be especially true of trials involving low socioeconomic groups where attrition rates may be greater than for other groups.

Studies involving interventions to support ‘abrupt’ smoking cessation report a wide range of attrition rates. In a review of RCTs of individual behavioural counselling interventions for smoking cessation [13], attrition rates (where reported) ranged from 1.7 % [14] to 22.4 % [15] at 6 months’ follow-up and from 3 % [16] to 31 % [17] at 12 months’ follow-up. A review of RCTs of interventions combining behavioural counselling and pharmacological support [18] identified a range of attrition rates from as low as 4–8 % [19] in one study and up to 24–30 % [20] in another at 6 months’ follow-up, and between 7 % [21] to 52 % [22] at 12 months’ follow-up in two other studies. Although one study identified in the review saw an attrition rate < 5 % at 24 months’ follow-up [23] it targeted inpatients with acute coronary syndrome who were probably more accessible for follow-up, compared with participants in the community. In contrast, a review of self-help interventions for smoking cessation [24], representing the least intensive level of intervention, included studies with attrition rates ranging from 11 % [25] to 66 % [26] at 6 months’ follow-up and <10 % [27] to 56 % [28] at 12 months’ follow-up.

Despite there being 60 systematic reviews on the Cochrane Database on the effectiveness of interventions for smoking cessation [4], little attention has been given to identifying the factors associated with study attrition. The factors associated with attrition in studies concerned with smoking reduction or involving disadvantaged smokers [29, 30] are particularly poorly understood, due to a small number of such studies.

A number of factors may influence attrition including: (i) the nature of the intervention (e.g., clinical trials of an investigational medicinal product (CTIMPs) versus clinical trials of complex behavioural interventions (non-CTIMPs); (ii) the population characteristics (e.g., socioeconomic status, demographics); (iii) the study design (e.g., length of time to follow-up, burden of data collection on participant); and (iv) specifically among smoking trials, a focus on abrupt smoking cessation versus smoking reduction.

Smoking reduction is increasingly recognised as a viable alternative to the traditional abrupt smoking cessation approach, with flexible outcome measures [31], and it is unclear if there is any difference between these approaches on attrition. A review comparing interventions involving smoking reduction or abrupt cessation [32] included ten studies with attrition rates ranging from 19.1 % [33] to 21–24 % [34] at 6 months, and 11–13 % [35] to 64 % [36] at 12 months but there appeared to be no difference in attrition between those reducing their smoking before quitting or stopping abruptly.

There have been no reports of when certain participants are likely to drop out, and given the progressive nature of smoking cessation and reduction it is likely certain participants may be more or less likely to withdraw at different times (i.e., time of dropout may be predicted by baseline characteristics such as confidence and importance to cut down or quit). Understanding these potential predictors of when a participant is likely to drop out would allow trialists to better plan resource use and direct support to participants at certain times to maximise the retention of participants.

Exercise as an aid to smoking cessation has been acknowledged as a feasible intervention for supporting cessation, yet the number of rigorous studies remains limited: there were only 15 studies included in the latest Cochrane review on the topic [37], 7 of which included fewer than 25 participants. Of the studies identified in this review, attrition rates varied from 0.3 % [38] to 60.8 % [39] at 6 months and from 0.5 % [40] to 68–75 % [41] at 12 months. The heterogeneity of research designs and methods among these studies makes it difficult to identify any factors associated with attrition, but attrition rates seem high compared with other studies involving interventions for smoking cessation.

Other studies which present attrition rates from smoking cessation studies with specific populations also present similar attrition rates to the smoking studies more generally. A study on exercise and counselling for smoking cessation for those with current depressive disorders reports attrition rates of 20 % and 37 % for the intervention and control respectively [42]. Another exercise counselling for smoking cessation among depressed women [43] reports slightly higher attrition rates of 35 % at 10 weeks with no difference between arms. A recent study using exercise to support pregnant smokers to quit reported 11 % attrition for unexplained reasons [44]. A study supporting homeless people to quit [45] reports 25 % attrition at 26 weeks with no difference between study arms. As with the broader smoking cessation literature, the reported cessation rates among specific populations are varied and within similar ranges, suggesting specific characteristics may not impact on overall attrition rates.

There are few studies on the effectiveness of interventions for smoking cessation among low socioeconomic groups. In a recent review of low-income groups and health-behaviour change interventions [46] only 13 studies were identified, and of those only 7 targeted smoking behaviour, and no studies examined potential predictors of and reason for study attrition. None of the identified studies focused on smoking reduction among low socioeconomic groups.

Some authors suggest that attrition rates are generally higher in the control condition [10–12], but in the literature we reviewed some studies showed greater attrition in the control or intervention arm, and the majority showed no difference between trial arms. A meta-analysis of a random sample of 100 randomised controlled trials (RCTs) published in medical journals (with a range of participants and interventions) also showed no differential attrition between intervention and control conditions [47].

Several factors have been associated with increased attrition in smoking trials among various populations, including a higher Fagerström Test for Nicotine Dependence (FTND) score [48], a lower intention to quit [49], low self-efficacy and a longer smoking history [25], and the number of cigarettes smoked per day [50–52]. It is commonly believed that attrition within smoking cessation studies is driven by failure to maintain a successful quit attempt, where the individual will no longer seek support once they have reinitiated smoking as it holds no value if they are smoking again. This could be why factors associated with failing to quit (such as level of addiction and dependence) are related to attrition. Less is known in relation to failure to reduce as a predictor of attrition.

The aim of this study is to identify the factors associated with participant attrition in a pilot RCT on the effectiveness of a novel Exercise-Assisted Reduction then Stop (EARS) intervention (HTA number 07/78/02, ISRCTN 13837944, UKCRN Study ID 8937) among disadvantaged smokers. EARS recruited a disadvantaged population assessed by predetermined criteria (e.g., 91 % were social class C2-E and 41 % indicated mental health problems), the details of which have been published elsewhere [53]. The specific objectives of this study are to determine if features of the trial design and methods, and participant characteristics, are associated with participant attrition, to inform the design and methods for a definitive trial.

## Methods

### Participants

Ethical approval for the study was granted by the National Health Service (NHS) National Research Ethics Service Committee South West, in the UK. Recruitment took place in the neighbourhoods of Devonport and Stonehouse (Plymouth, UK), which are among the 3 % most deprived areas in the UK. The recruitment methods, factors influencing recruitment, and baseline characteristics of the sample, have been reported elsewhere [54]. In summary, 99 adult moderate to heavy smokers, who wanted to reduce smoking (without nicotine replacement therapy (NRT)) but had no plans to quit in the next month, were recruited by either a mailed invitation from their general practitioner or NHS Stop Smoking Services (SSS), with follow-up telephone calls, or through other community approaches.

### Procedures

After providing informed consent and baseline information, participants were randomised to receive either usual care (consisting of brief advice on smoking cessation services) or usual care plus the EARS intervention (consisting of up to 12 weekly client-centred individual support sessions, via telephone or in person, to assist with making self-directed changes in smoking and physical activity behaviour). Participants in either arm of the trial expressing the desire to quit were offered the chance to be referred to local SSS for specialist support.

Follow-up assessments were completed at 4, 8, and 16 weeks post-randomisation. For those who missed follow-up appointments, up to five attempts were made by telephone to reschedule the appointment; the rescheduled appointment could take place up to the halfway point between the missed appointment and the next follow-up. After the halfway point attempts were made to schedule the next follow-up appointment. Those who could not be contacted at all were classified as having dropped out of the study. Reasons for withdrawal were recorded for those who explicitly withdrew consent to participate in the study.

### Measures

At baseline the following data were collected: participant demographic information (i.e., age, sex, marital status, cohabiting with other smokers, parental status (single parent living with a dependent under 16 years of age), employment status (employed or not), job status (social class), age of leaving full-time education, ethnicity, weight, and height), smoking history (age participant started smoking, longest period of cessation in the last year, attempts at cutting down, cessation aids used in the past year, use of SSS), number of cigarettes being smoked per day, Fagerström Test for Nicotine Dependence (FTND; [55, 56]) scores, stage of readiness to use physical activity to control smoking behaviour, expired air carbon monoxide (CO), and physical activity data (subjectively by self-report of the previous 7 days and objectively by accelerometer). Follow-up assessments captured data on smoking- and physical activity-related behaviours and attitudes. In order to ensure compliance with wearing and returning accelerometers (costing approximately £250 each) we initially paid participants £10 for returning the accelerometer at each time point (except week 4 when they were not worn). This was increased to £30 at each time point when it was observed a considerable number had not been returned about a third of the way through the study. No other payment was made to participants for completing assessments other than reimbursing travel expenses. For the purposes of the present study, those lost to follow-up before the final follow-up (week 16) was the primary binary outcome. We also classified participants as dropping out early or late: early dropouts were those who did not complete any assessment after baseline, and late dropouts were those who failed to complete follow-up assessments after week 4. Field notes were maintained to capture qualitative reasons for attrition as reported by withdrawing participants and as observed by researchers.

### Data analysis

To determine the factors associated with study attrition at 16 weeks, binary logistic regression was performed and odds ratios (OR) reported with 95 % confidence intervals (95 % CI). Intervention arm, method of recruitment, participant demographics, and lifestyle, behavioural, and attitudinal characteristics were individually examined as determinants of attrition, based on existing literature and the researchers’ a priori reasons for their inclusion. Following the univariable analyses, each significant predictor of attrition was then added to multivariable logistic regression. Further exploratory analysis sought to compare those who dropped out earlier (before week 4), later (after week 4), or completed the study. Attrition status (early dropout, late dropout or completion) was analysed using multinomial logistic regression, with inclusion of each covariate individually in a separate univariable model, followed by the inclusion of the variables related to attrition in a multivariable multinomial logistic regression model. Multinomial logistic regression was chosen in place of ordinal logistic regression as the three categories were considered to be qualitatively different and not necessarily sequential.

Additional exploratory analyses examined change in cigarettes smoked per day (and therefore the success of individual change) as a predictor of dropout. For those who were followed up at least once post baseline, two categorical variables of at least a 50 % reduction and any positive reduction from baseline to week 4 and week 8 were coded and analysed through univariable binary logistic regression in relation to drop out before week16.

All statistical analyses were completed using Stata SE (v. 12.0) (StataCorp LP, College Station, TX, USA).

## Results

The overall sample characteristics for the 99 participants recruited and randomised have been reported elsewhere [49]. Data were collected from 61.6 % (*n* = 61) at 16 weeks post baseline. Study attrition occurred primarily soon after baseline with 21 of the 38 participants lost to follow-up not completing the week 4 assessment.

Table 1 shows that for the sample as a whole those with high self-reported confidence to quit in the next 6 months (*n* = 48) were less likely to be lost to follow-up than those with low confidence. Also, those completing at least 150 mins of moderate and vigorous physical activity (MVPA) per week (*n* = 69) were also less likely to be lost to follow-up.

**Table 1:** Summary of logistic regression analysis for study dropout versus completion

	Variable	Number	Odds ratio (95 % CI)
Methods	Trial arm^a^		
	Intervention	49	1.03 (0.46; 2.32)
	Recruitment avenue^b^		
	Stop Smoking Services	31	0.81 (0.33; 1.99)
	Community	6	0.74 (0.13; 4.35)
	Recruitment method^c^		
	Letter plus telephone reminder	38	2.24 (0.95; 5.26)
	Community	6	1.12 (0.19; 6.70)
Demographics	Age (years)	99	0.97 (0.93; 1.01)
	Gender^d^		
	Female	56	1.55 (0.68; 3.56)
	Body mass index	98	1.01 (0.95; 1.07)
	Employment status^e^		
	Unemployed	45	1.13 (0.50; 2.55)
	Job status^f^		
	C2-D	45	1.21 (0.27; 5.50)
	Unemployed	45	1.33 (0.29; 6.03)
	Age left education	99	0.93 (0.73; 1.17)
Smoking-related variables	Years smoking	99	0.98 (0.94; 1.01)
	Previous use of Stop Smoking Services^g^		
	Have not used Stop Smoking Services in the past	58	1.14 (0.50; 2.60)
	Cigarettes per day	99	1.00 (0.97; 1.03)
	Expired air carbon monoxide (parts per million)	98	1.04 (0.99; 1.09)
	Fagerström Test for Nicotine Dependence	99	1.19 (0.96; 1.46)
	Importance of quitting next 6 months (median)^h^		
	High importance	49	1.23 (0.54; 2.76)
	Confidence to quit in the next 6 months (median)^i^		
	High confidence	48	0.43 (0.19; 0.99)
	Confidence to cut down by half in the next month (median)^j^		
	High confidence	39	1.44 (0.63; 3.28)
Physical activity-related variables	Self-reported ≥30 mins of moderate and vigorous physical activity per day^k^		
	Yes	65	0.48 (0.20; 1.12)
	Self-reported minutes of moderate and vigorous physical activity per day	99	1.00 (0.99; 1.00)
	Self-reported ≥30 mins moderate and vigorous physical activity on at least 5 days^l^		
	Yes	43	0.77 (0.34; 1.75)
	Self-reported ≥150 mins moderate and vigorous physical activity per week^m^		
	Yes	69	0.33 (0.14; 0.81)
	Accelerometer ≥30 mins moderate and vigorous physical activity per day^n^		
	Yes	32	1.11 (0.42; 2.95)
	Accelerometer minutes moderate and vigorous physical activity per day	66	1.00 (0.98; 1.02)
	Stage of change to use physical activity to control smoking^o^		
	Planning, action, maintenance	20	0.42 (0.15; 1.40)
	Confidence to exercise for ≥30 mins on most days over next 6 months^p^		
	High confidence	55	0.83 (0.37; 1.86)
	Confidence to walk for ≥15 mins at a brisk pace^q^		
	High confidence	59	1.06 (0.47; 2.43)
	Indicated mental health problem^r^		
	Yes	41	1.49 (0.65; 3.38)

^a^Reference: control; ^b^Reference: primary care; ^c^Reference: letter only; ^d^Reference: male; ^e^Reference: employed; ^f^Reference: social class A–C1; ^g^Reference: have used SSS in the past; ^h^Reference: low importance; ^i^Reference: low confidence; ^j^Reference: low confidence; ^k^Reference: not reporting 30 mins MVPA per day; ^l^Reference: not completing 30 mins MVPA on at least 5 days per week; ^m^Reference: not reporting >150 mins MVPA per week; ^n^Reference: not completing 30 mins MVPA per day as assessed by accelerometer; ^o^Reference: pre-contemplation and contemplation; ^p^Reference: low confidence; ^q^Reference: low confidence; ^r^Reference group: no indicated mental health problem

The multivariable binary logistic regression of variables found to be related to attrition in the univariable analyses Revealed that in the presence of other variables, only the completion of 150 mins of moderate and vigorous physical activity (MVPA) per week or more was related to a lower odds ratio of being lost to follow-up (OR (95 % CI) 0.32 (0.13; 0.80)). Confidence to quit in the next 6 months was not related to lower odds of being lost to follow-up (OR (95 % CI) 0.43 (0.18; 1.03)).

Table 2 presents descriptive data of the continuous variables between early dropouts, late dropouts and completers. Age, confidence to quit, and smoking history appeared to vary by withdrawal status; younger people, those with lower confidence to quit in the next 6 months, and those with shorter smoking history seemed more likely to drop out early. There also appeared to be a trend for those dropping out early in the study to have left education later and to have reported lower baseline expired air CO value than those dropping out later.

**Table 2:** Comparison of continuous baseline variables by early dropouts, late dropouts, and study completers

	Number	Early dropout (before week 4)	Late dropout (after week 4)	Completer
Age; mean (SD), *n*	99	40.9 (10.2), 21	48.2 (10.7), 17	48.1 (11.4), 61
Age left education; mean (SD); median (IQR), *n*	99	16.5 (1.3); 16 (16; 17), 21	15.7 (0.9); 15 (15; 16), 17	16.4 (2.2); 16 (15;16)
BMI; mean (SD); median (IQR), *n*	98	29.0 (7.9); 28.0 (23.2; 33.3), 21	27.5 (5.7); 27.3 (22.1; 32.6), 17	28.0 (6.0); 27.0 (22.5; 31.7), 60
Years smoking; mean (SD); median (IQR), *n*	99	25.3 (11.9); 27.7 (12.6; 31.8), 21	35.2 (11.6); 37.4 (24.6; 44.7), 17	33.3 (11.9); 35.8 (23.4; 43.1), 61
CPD; mean (SD); median (IQR), *n*	99	19.2 (7.6); 19.8 (13.3; 27.8), 21	23.7 (21.2); 19.6 (15.0; 27.8), 17	21.8 (13.8); 18.9 (15.0; 23.9), 61
CO; mean (SD); median (IQR), *n*	98	17.9 (10.0); 14 (11; 21), 21	21.6 (5.2); 21.5 (17.5; 24), 16	17.1 (7.8); 16 (12; 22), 61
FTND; mean (SD), *n*	99	5.7 (1.8), 21	6.4 (2.2), 17	5.3 (2.1), 61
Self-reported MVPA per day; mean (SD); median (IQR), *n*	99	72.3 (91.0); 42.1 (0; 109.3), 21	43.33 (49.3); 34.3 (0; 111.4), 17	81.0 (98.6); 47.1 (77.1; 25.7), 61
Accelerometer MVPA; mean (SD); median (IQR), *n*	66	29.3 (20.4); 27.6 (13.2; 42.5), 19	41.3 (32.8); 35.3 (16.3; 44.9), 9	31.0 (24.3); 25.8 (11.8; 43.7), 38
Importance of quitting in next 6 months; mean (SD); median (IQR), *n*	99	5.4 (1.5); 6 (4;7), 21	5.1 (2.0); 6 (4;7), 17	5.3 (1.7); 5 (5; 7), 61
Confidence to quit in next 6 months; mean (SD), *n*	97	3.1 (1.4), 21	2.7 (1.5), 17	3.9 (1.7), 59

*SD* standard deviation, *IQR* interquartile range, *BMI* body mass index, *CPD* cigrettes per day , *CO* carbon monoxide, *FTND* Fagerström Test for Nicotine Dependence, *MVPA* moderate and vigorous physical activity

The odds of participant dropout late in the study (versus completion) were increased for those recruited via follow-up telephone calls (Table 3). Greater confidence to quit in the next 6 months was associated with lower odds of late dropout versus completion compared with lower confidence. With increasing age, the odds of early dropout versus completion were reduced, but age did not appear to be associated with odds of late dropout versus completion; years of smoking showed a similar association with both early and late dropout versus completion. Those who reported doing 150 mins or more of MVPA per week at baseline had lower odds of early dropout compared with participants who did not complete at least 150 mins of MVPA per week; however, no equivalent association was found with regard to late dropout.

**Table 3:** Summary of multinomial logistic regression analysis for study completion status: late/early dropout versus completion

		Early dropouts (before week 4)		Late dropouts (after week 4)	
	Variable	Number	Odds ratio (95 % CI)	Number	Odds ratio (95 % CI)
	Trial arm^a^				
	Intervention	9	0.78 (0.29; 2.10)	10	1.43 (0.50; 4.39)
Recruitment	Recruitment avenue^b^				
	Stop Smoking Services	6	0.79 (0.26; 2.39)	5	0.84 (0.26; 2.77)
	Community	1	0.66 (0.07; 6.42)	1	0.84 (0.08; 8.33)
	Recruitment method^c^				
	Telephone	9	1.63 (0.58; 4.62)	10	3.32 (1.05; 10.60)
	Community	1	0.86 (0.87; 8.58)	1	1.58 (0.15; 16.61)
Demographics	Age (years)	21	0.94 (0.90; 0.99)	17	1.00 (0.95; 1.05)
	Gender^d^				
	Female	13	1.48 (0.53; 5.05)	11	1.67 (0.54; 5.05)
	Body mass index	21	1.02 (0.95; 1.11)	17	0.99 (0.33; 1.08)
	Employment status^e^				
	Unemployed	7	0.63 (0.22; 1.79)	11	2.32 (0.76; 7.03)
	Job status^f^				
	C2-E	13	2.77 (0.30; 25.53)	4	0.43 (0.06; 2.92)
	Unemployed	7	1.55 (0.16; 15.18)	11	1.22 (0.21; 7.03)
	Age left education	21	1.04 (0.82; 1.31)	17	0.64 (0.36; 1.14)
Smoking history	Years smoking		0.95 (0.90; 0.99)		1.16 (0.97; 1.06)
	Previous use of Stop Smoking Services^g^				
	No	14	01.49 (0.53; 4.22)	9	0.84 (0.28; 2.46)
Smoking-related variables	Cigarettes per day	21	0.82 (0.94; 1.03)	17	1.01 (0.97; 1.04)
	Expired air carbon monoxide (parts per million)	21	1.01 (0.95; 1.08)	16	1.07 (1.00; 1.14)
	Fagerstrom Test for Nicotine Dependence	21	1.01 (0.86; 1.42)	17	1.31 (0.98; 1.73)
	Importance of quitting in the next 6 months (median)^h^				
	High 6–7	11	1.21 (0.45; 3.29)	9	1.24 (0.42; 3.63)
	Confidence to quit in the next 6 months (median)^i^				
	High 4–7	12	0.55 (0.20; 1.51)	10	0.31 (0.95; 0.98)
	Confidence to cut down by half in the next month (median)^j^				
	High 5–7	3	1.95 (0.71; 5.31)	2	0.97 (0.31; 2.97)
Physical activity- related variables	Self-reported ≥30 mins of moderate and vigorous physical activity per day^k^				
	Yes	11	0.42 (0.15; 1.19)	10	0.55 (0.18; 1.68)
	Self-reported minutes of moderate and vigorous physical activity per day	21	1.00 (0.99; 1.00)	17	0.99 (0.98; 1.00)
	Self-reported ≥30 mins of moderate and vigorous physical activity on at least 5 days^l^				
	Yes	9	0.89 (0.33; 2.41)	6	0.64 (0.21; 1.95)
	Self-reported ≥150 mins of moderate and vigorous physical activity per week^m^				
	Yes	11	0.30 (0.10; 0.85)	10	0.39 (0.12; 1.21)
	Accelerometer ≥30 mins of moderate and vigorous physical activity per day^n^				
	Yes	8	0.81 (0.27; 2.46)	6	2.23 (0.48; 10.18)
	Accelerometer total minutes of moderate and vigorous physical activity per day	19	1.00 (0.97; 1.02)	9	0.54 (0.99; 1.04)
	Stage of change to use physical activity to control smoking^o^				
	Planning, action, maintenance	2	0.32 (0.07; 1.55)	3	0.66 (0.17; 2.61)
	Confidence to exercise for ≥30 mins on most days over next 6 months^p^				
	High (6–7)	12	0.99 (0.36; 2.69)	8	0.66 (0.23; 1.93)
	Confidence to walk for ≥15 mins at a brisk pace^q^				
	High (7)	12	0.46 (0.34; 2.53)	11	1.27 (0.41; 3.90)
	Indicated mental health problem^r^				
	Yes	11	1.82 (0.67; 4.95)	7	1.16 (0.39; 3.46)

^a^Reference: control; ^b^Reference: primary care; ^c^Reference: letter only; ^d^Reference: male; ^e^Reference: employed; ^f^Reference: social class A-C1; ^g^Reference: have used SSS in the past; ^h^Reference: low importance; ^i^Reference: low confidence; ^j^Reference: low confidence; ^k^Reference: not reporting 30 mins MVPA per day; ^l^Reference: not completing 30 mins MVPA on at least 5 days per week; ^m^Reference: not reporting >150 mins MVPA per week; ^n^Reference: not completing 30 mins MVPA per day as assessed by accelerometer; ^o^Reference: pre-contemplation and contemplation; ^p^Reference: low confidence; ^q^Reference: low confidence; ^r^Reference group: no indicated mental health problem

Variables shown to be related to attrition in the univariable multinomial analyses were carried forward into a multivariable multinomial analysis and are show in Table 4. Only the completion of 150 mins of MVPA per week or more retained any significance in the presence of the other variables, with those completing more than 150 mins of MVPA per week at baseline being less likely to drop out early than later in the study when compared to study completers.

**Table 4:** Multivariable multinomial logistic regression for study completion status: late/early dropout versus completion (*N* = 97)

	Early dropouts (before week 4)		Late dropouts (after week 4)	
Variable	Number	Odds ratio (95 % CI)	Number	Odds ratio (95 % CI)
Self-reported ≥150 minutes of moderate and vigorous physical activity per week^m^				
Yes	11	0.23 (0.07; 0.75)	10	0.52 (0.14; 1.90)
Confidence to quit in the next 6 months (median)^i^				
High (4–7)	9	0.50 (0.16; 1.51)	5	0.37 (0.11; 1.27)
Recruitment method^c^				
Telephone	9	1.25 (0.39; 4.01)	10	2.77 (0.79; 9.78)
Community	1	0.51 (0.42; 6.17)	1	1.72 (0.14; 21.33)
Age (years)	21	1.00 (0.85; 1.17)	17	0.84 (0.65; 1.06)
Years smoking	21	0.94 (0.81; 1.09)	17	1.21 (0.95; 1.54)

^a^Reference: not reporting >150 minutes MVPA per week; ^b^Reference: low confidence; ^c^Reference: letter only

Exploratory analyses of change in cigarettes smoked per day (from baseline to either week 4 or week 8), shown in Table 5, showed no significance in predicting study dropout before week 16.

**Table 5:** Logistic regression of study attrition for change in cigarettes smoked per day before week 16 (*N* = 78) (late dropout versus completion)

Variable	Number	Odds ratio (95 % CI)
Reduction of 50 % or more before week 16		
Yes	28	0.31 (0.08; 1.19)
Any reduction in cigarettes smoked per day before week 16		
Yes	58	0.55 (0.17; 1.74)

Qualitative reasons for dropout were not possible to obtain directly from participants whom we were unable to contact. Of those who explicitly withdrew consent (*n* = 15), the reasons for dropout included illness or death of a close family member, advice from a mental health care worker that the participant had become anxious about involvement in the study, time pressures elsewhere, expecting a greater financial reward for taking part (indicating a possible misunderstanding of study procedures due to poor explanation), and being dissatisfied with allocation to the control condition.

## Discussion

The overall attrition rate of 38.4 % at 16 weeks falls within the range of attrition rates identified in other broader trials of smoking cessation. In the absence of similar studies, the overall retention in this study could be regarded as acceptable for a group of disadvantaged smokers and provides valuable information for a larger study. Unlike some trials, we did not explicitly pay participants to complete follow-up assessments and one may assume a lower attrition rate had we done so.

The fact that over 50 % of those dropping out did so before week 4 suggests that particular focus is needed on new ways to maintain participation in the initial stages of trial engagement. Although attrition in both treatment arms was the same in the present study, it may be that the predictors of attrition may vary between arms. However, the numbers in this pilot trial were insufficient to inferentially test this hypothesis.

The only trial design factors to influence attrition was whether or not participants were recruited by follow-up telephone call; those recruited by this more intensive approach were more likely to drop out later than earlier in the study, possibly reflecting ambivalence to the invitation. We deliberately conducted follow-up telephone calls to recruit smokers in case they had low literacy levels. It may be that providing further data after baseline was too challenging and we should consider providing more support to keep these individuals in the study. Recruitment via different locations (primary care versus SSS), and the method of recruitment, also showed no effect on attrition in the sample as a whole. We found equal attrition in both the intervention and control arms, which has been reported elsewhere [43].

The mean age of those dropping out early in the study was younger than the mean age of those completing the study. Age has been reported elsewhere to predict attrition, with older participants less likely to drop out [57], suggesting they may be more committed and able to have more time to remain in a trial [1]. This highlights the need for additional support for engaging younger people. However, other studies have not found age to be related to attrition [9, 50]. No other participant demographic characteristics showed evidence of any relationship with study attrition. Our researchers worked flexible hours to conduct assessments with participants (in employment or not), and this may have reduced the risk of attrition.

We were interested in whether we could retain more dependent and heavier smokers in the trial. The finding that those with a longer smoking history and a trend for smoking more cigarettes were more likely to complete the study was encouraging. They may suggest that a trial focused on cutting down may be more appealing to heavier smokers, a finding reported elsewhere [58]. In contrast it appeared that those with greater confidence to quit (normally associated with lower dependence) were less likely to withdraw, specifically, less likely to withdraw later in the study. These preliminary contrasting findings are not easy to explain but it would appear that future researchers should stress that smoking behaviour and related beliefs (such as confidence to quit) should not influence continued participation in a trial.

In smoking cessation studies, smoking relapse is typically associated with attrition and, similarly, it may be that a failure to reduce smoking levels is associated with attrition. Also, several of the variables shown to predict attrition in the present study are the same as those that predict smoking relapse (e.g. low self-efficacy, lower age) and there may be a common set of variables that predict smoking relapse, failure to reduce and attrition. There may also be variables that are specific to smoking reduction versus cessation studies. For example, a higher level of cigarette dependence reliably predicts smoking relapse [59] but in the current study higher dependence was associated with less attrition.

Those who completed at least 150 mins of MVPA per week at baseline were also less likely to withdraw than those reporting less activity; specifically, these participants were less likely to withdraw early in the study. This finding remained significant even in the presence of other predictor variables in the multivariable model. This replicates the findings from another study in which those who were inactive at baseline were significantly more likely to drop out of an arm of a trial with a focus on fitness training [60]. Greater study attrition among less active participants in a physical activity study has the potential to reduce the size of effects due to a ceiling effect. It also poses a threat to external validity if the findings cannot be generalised to less active populations. Other research involving low socioeconomic groups on the effectiveness of a physical activity intervention [60] reported that attrition rates were significantly higher in those randomised to a ‘fitness assessment’ intervention compared with an ‘exercise consultation’ intervention. This suggests that intervention content may differentially influence attrition. The present study involved physical activity (PA) counselling (as opposed to an emphasis on ‘fitness’) and this may have helped to increase study retention. Avoiding an emphasis on ‘fitness’ may have helped to maximise external validity by engaging with and retaining both those who are and are not already physically active. However, the current study engaged with a comparatively active sample (potentially due to self-selection), meaning analyses may be less likely to show an effect (due to a ceiling effect). It also limits the application of the findings to a more general, less active, population. The levels of self-reported physical activity were not corroborated by objective activity measurement at baseline (accelerometer) and due to the low numbers and variance in self-reported physical activity, more research is needed to further explore if baseline physical activity influences study attrition.

Failure or success to reduce the amount of cigarettes smoked between baseline and week 8 showed no confidence in predicting dropout before week 16. This is likely limited by the lack of precision due to the small sample size, as the trend was in favour of those who achieved a reduction in cigarettes smoked before week 16 to demonstrate lower odds of withdrawal, as might be expected.

The present exploratory study had several limitations. Due to the relatively low numbers involved in this pilot trial (and as a result the low number of observations of the outcome of interest), some caution should be used in interpreting the findings due to their imprecision. Nevertheless, we have identified how the findings may influence planning a larger study, and further such analysis should be considered in any future larger study to estimate bias from missing data and study attrition.

The study was also limited in the ethnic diversity of the sample, with 97 % reporting being white British, which is typical of the geographical area in which the study was located. This limits the findings to other more ethnically diverse populations and is something that would need to be considered carefully in future research (as ethnicity is something that has been found to be predictive of dropout in other studies [1]).

We chose not to incentivise data capture to avoid the potential of influencing trial outcomes in this pragmatic study. Preliminary work also revealed that financial incentive was treated with caution by some participants for fear of jeopardising government unemployment benefits. The payment for returning an accelerometer was implemented in an attempt to minimise the loss of expensive equipment and not as an incentive for participation, although it may have acted as such. Incentivisation could be considered for future research in more detail.

## Conclusions

The present research provides important information on factors that may influence attrition within a multi-component smoking reduction study among low socioeconomic status smokers. Retention was at least comparable with the few other studies involving disadvantaged groups with smoking behaviour as a main outcome. These analyses provide unique information on retention in a study aimed at smokers in these groups who did not wish to quit. Only a few factors were quantitatively associated with attrition, suggesting that further research is needed to explain why participants in this type of study drop out. Qualitatively, the diverse reasons for study attrition appeared to be mainly due to unpredictable participant life events and perhaps a misunderstanding about trial involvement.

## Abbreviations

CO: Carbon monoxide
CTIMP: Clinical trial of an investigational medicinal product
EARS: Exercise-Assisted Reduction then Stop
FTND: Fagerström Test for Nicotine Dependence
HTA: Health Technology Assessment
ISRCTN: International Standardised Randomised Controlled Trial Number
ITT: Intention to treat
MVPA: Moderate and vigorous physical activity
NHS: National Health Service
NRT: Nicotine replacement therapy
PA: Physical activity
RCT: Randomised controlled trial
SSS: Stop smoking services
UKCRN: United Kingdom Clinical Research Network
